# Recyclable Hydrotalcite-Supported Copper Catalysts for Green and Regioselective Click Synthesis of 1,2,3-Triazoles

**DOI:** 10.1021/acsomega.5c10849

**Published:** 2026-01-27

**Authors:** Gustavo S. G. de Carvalho, Douglas C. A. Pinto, Fernando de C. da Silva

**Affiliations:** Instituto de Química, Departamento de Química Orgânica, 28110 Universidade Federal Fluminense, Campus do Valonguinho, CEP, Niterói 24020-141, RJ, Brazil

## Abstract

This work reports a sustainable and highly efficient catalytic system based on cuprous oxide nanoparticles supported on hydrotalcite (**Cu**_**2**_ **O@LDH-01**) for the regioselective synthesis of 1,2,3-triazoles via copper-catalyzed azide–alkyne cycloaddition (CuAAC) in aqueous media. The catalyst was comprehensively characterized by XRD, FTIR, BET, TGA, and STEM-EDX analyses, confirming the successful incorporation and uniform dispersion of the Cu­(I) species within the layered double hydroxide structure. Under mild and additive-free conditions, **Cu**_**2**_ **O@LDH-01** afforded quantitative conversions in water within only 5 min under ultrasound irradiation, emphasizing the crucial role of acoustic cavitation in enhancing mass transfer and catalytic performance. The system exhibited regioselectivity across a broad range of alkynes and azides, with both electronic and steric factors governing reactivity. Furthermore, the catalyst could be readily recovered and reused over five consecutive cycles without a noticeable loss of activity, demonstrating its robustness and practical applicability. Overall, this study highlights hydrotalcite-supported Cu­(I) catalysts as a green, recyclable, and scalable platform for click chemistry, in full alignment with the principles of sustainable synthesis and waste minimization.

## Graphical abstract

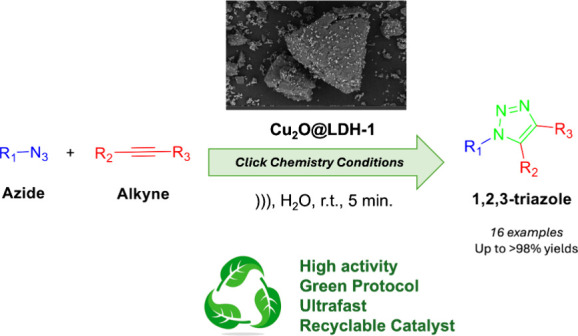

## Introduction

The Huisgen cycloaddition between azides and alkynes, classically a slow and nonselective 1,3-dipolar reaction, was revolutionized by the advent of copper catalysis (CuAAC), which enables the efficient and regioselective synthesis of 1,4-disubstituted and 1,2,3-triazoles under mild conditions.^1^ These nitrogen-rich heterocycles occupy a pivotal position in pharmaceutical, materials, and agrochemical chemistry, serving as versatile motifs in ligands, drugs, biomarkers, bioactive molecules, and functional components of advanced polymeric materials.^2^ However, conventional homogeneous catalytic systems face inherent drawbacks, including challenging catalyst recovery, contamination of products with residual metal ions, and limited recyclability, all of which hinder their large-scale and sustainable application.^1,3^

In this context, the heterogenization of Cu­(I)-based catalysts^4,5^ has emerged as a strategic approach that combines high catalytic activity with facile separation and reusability.^6–7^ Among the various solid supports explored, systems such as Cu@KCC-1-NH–CS_2_,^8^ CuNPs@HT,^9^ Cu­(I)-MOFs,^10^ and magnetic ferrite nanoparticles^11^ have demonstrated the potential of well-structured materials in promoting azide–alkyne cycloadditions. The catalytic performance of supported Cu­(I) nanoparticles in the CuAAC reaction provides valuable insights into the intrinsic chemistry of the substrates. As highlighted by Gawande et al.,^12^ copper displays remarkable versatility in catalysis due to its ability to access multiple oxidation states (Cu°, Cu^I^, Cu^II^), thus enabling both one- and two-electron redox pathways. This redox flexibility plays a central role in the activation of alkynes during CuAAC, particularly in heterogeneous catalytic systems such as that investigated herein.

In particular, the use of Cu_2_O as the active phase offers several notable advantages, including high stability, low cost, low toxicity, and favorable electronic properties.^2,12^ When synthesized with morphological control such as cubic nanoparticles with an average size of approximately 7.5 nm, Cu_2_O exhibits a high surface area, a reduced band gap (∼2.0 eV), and a low tendency toward leaching, all critical factors for its sustainable catalytic application. Moreover, its solid nature minimizes product contamination and enables efficient recovery and reuse, thereby overcoming the main limitations of homogeneous systems.^12,13^ However, the intrinsic catalytic performance of any active phase depends critically on the effective stabilization and uniform dispersion of its active sites, making the selection of an appropriate support a decisive factor in the design of robust heterogeneous catalysts.

Hydrotalcite (HT), with its characteristic lamellar structure and tunable interlayer spacing (2–5 nm), provides an ideal environment for anchoring and stabilizing Cu_2_O nanoparticles, preventing aggregation, and promoting a homogeneous distribution of active sites. Thus, beyond its general utility as a support, the inherent basicity and stabilizing lamellar structure of hydrotalcite provide a uniquely synergistic environment for immobilizing and activating Cu­(I) species specifically for the CuAAC reaction.^9,15–17^ Once the active Cu­(I) species, system robustness, and optimal reaction conditions are established, variations in yield primarily arise from the molecular characteristics of the reaction partners. The choice of hydrotalcite as a support is therefore deliberate: as emphasized by Gawande et al.,^12^ layered materials such as hydrotalcites offer confined sites that stabilize metal nanoparticles, maintaining their dispersion and accessibility, essential features for high catalytic efficiency.^14,17^ This property is particularly important for sustaining high activity, even in aqueous media. Additionally, the intrinsic basicity of hydrotalcite (pH ≈ 10) facilitates deprotonation of the terminal alkyne, a key step in the CuAAC mechanism, often eliminating the need for external bases.^18^ HT is also compatible with green solvents such as water, thermally stable (up to ∼400 °C), nontoxic, and recyclable, features that align well with the core principles of green chemistry.^15^

To further advance these heterogeneous catalytic systems, ultrasound activation has emerged as a powerful and sustainable strategy. The presence of Lewis acidic sites facilitates the reaction,^15^ while the acoustic cavitation generated by ultrasonic irradiation significantly enhances mass transfer at the solid–liquid interface and produces localized heating, thereby accelerating the reaction without a substantial increase in the bulk temperature.^20–23^ This physical activation prevents nanoparticle agglomeration,^13^ preserves the accessibility of catalytic sites, and eliminates the need for reducing additives such as sodium ascorbate or even an inert atmosphere.^22^ The resulting synergy between hydrotalcite-supported Cu_2_O and ultrasonic irradiation establishes a new paradigm for the sustainable, efficient, and scalable synthesis of 1,2,3-triazoles.

Recent studies have demonstrated that this strategy allows reactions that traditionally required several hours to be completed within minutes, affording high yields, excellent selectivity, and catalyst reusability.^23,24^ While Yu et al.^23^ reported Cu­(I) stabilization through electronic interactions with acid-doped graphdiyne (GDY), Zhang et al.^24^ explored plasmonic effects in Au@Cu_2_O as a photothermal alternative for tuning catalytic reactivity. However, ultrasound-assisted catalysis represents a more accessible and environmentally benign approach compared with photoactive systems that rely on specialized light sources.

In this context, the present work introduces a catalytic system based on heterogenized and ultrasound-activated Cu_2_O supported on hydrotalcite (HT) for the efficient synthesis of 1,2,3-triazoles. The study aims to establish correlations among catalyst structure, irradiation parameters, and catalytic performance, focusing not only on the synthetic efficiency but also on the practical and industrial feasibility of the process. Ultimately, this work seeks to overcome the limitations of conventional methodologies and advance toward a robust, economical, and environmentally responsible catalytic platform.

## Results and Discussion

### Catalyst Synthesis and Characterization

Layered double hydroxides (LDHs), hereafter referred to as **LDH-01** (Mg/Al) and **LDH-02** (Mg/Cu/Al), were synthesized by the coprecipitation method according to De Carvalho et al. (2019).^25^ The supported nanocatalyst **Cu**_**2**_ **O@LDH-01** was prepared in aqueous medium using hydrazine hydrate as the reducing agent and copper­(II) sulfate as the copper source, with **LDH-01** serving as the support.^9,26^ The resulting materials were characterized by X-ray diffraction (XRD), Fourier transform infrared (FTIR) spectroscopy, thermogravimetric analysis (TGA), scanning electron microscopy (SEM), and energy-dispersive X-ray spectroscopy (EDX) analyses in accordance with procedures reported in the literature.

The XRD patterns of **LDH-01** and **LDH-02** are shown in Figure 1. Both samples exhibit the characteristic reflections of hydrotalcite-like LDHs, confirming the formation of well-crystallized materials with a single LDH phase. The diffractograms display the typical basal reflections at 2θ ≈ 11.9°, corresponding to the (003) plane, along with higher-order reflections at (006), (009), (015), (018), (110), and (113).^25,27^ The powder XRD pattern of **Cu**_**2**_ **O@LDH-01** reveals, in addition to the LDH reflections, several additional peaks assigned to the Cu_2_O phase. These are primarily characterized by the reflection at 2θ = 36.56°, corresponding to the (111) plane, as well as additional reflections at (110), (200), and (220), confirming the successful formation of the supported Cu_2_O nanoparticles.^26,28^

**Figure 1:**
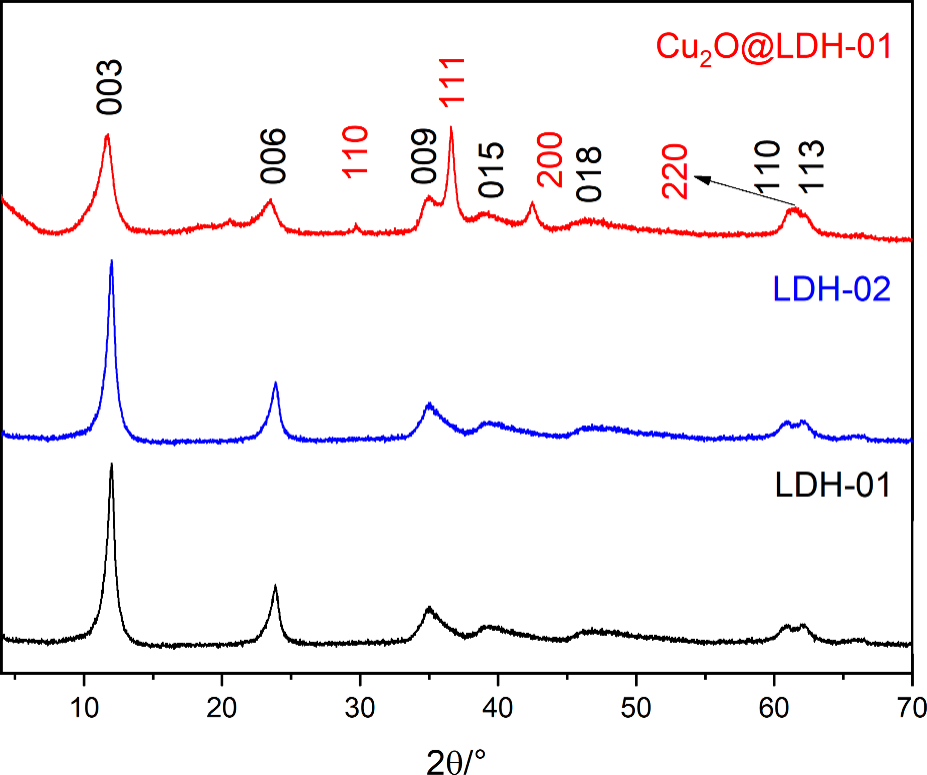
Powder XRD patterns of **LDH-01**, **LDH-02**, and **Cu**_2_ **O@LDH-01**. The Miller indices for the characteristic reflections are indicated: **black** for the LDH phase and red for the Cu_2_O phase.

The FTIR spectra of the samples exhibit broad absorption bands, characteristic of the interlayer and adsorbed water molecules within the LDH structure (Figure 2). The broad band in the 3000–3700 cm^–1^ region corresponds to the O–H stretching (ν_OH_) vibrations, while the band at approximately 1635 cm^–1^ is assigned to the H–O–H bending (δ_OH_) mode of water. The strong absorption near 1360 cm^–1^ is attributed to the asymmetric stretching vibration of interlayer carbonate ions, consistent with the interaction between carbonate and water that gives rise to the broad feature between 1300 and 1560 cm^–1^ due to symmetry reduction of the carbonate species.^27^ Additional bands observed at 770–790 and 685 cm^–1^ correspond to the out-of-plane and in-plane bending modes of CO_3_^2–^, respectively, confirming the presence of carbonate ions within the LDH interlayers.^25^

**Figure 2:**
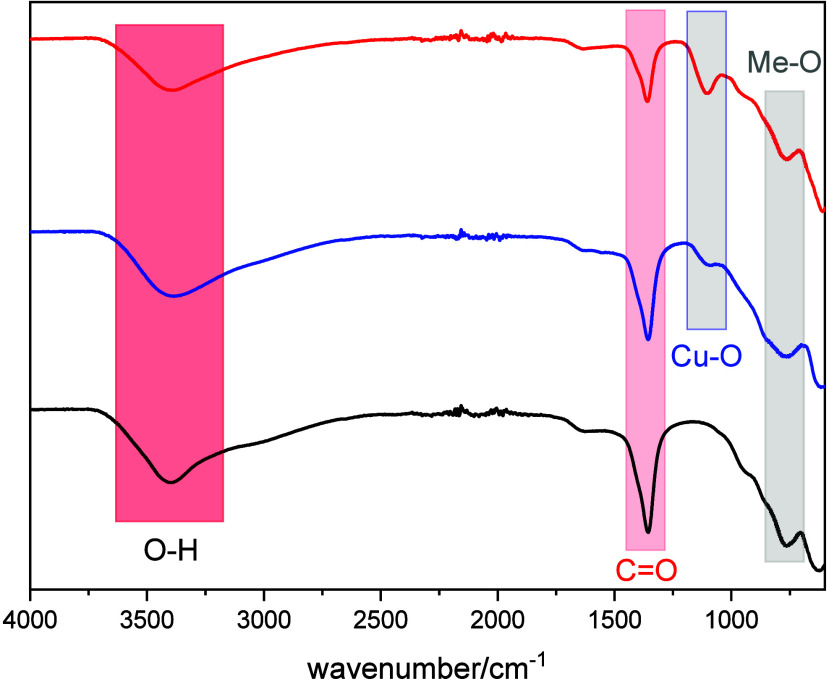
FTIR spectra of the catalysts and support (**black:** **LDH-01**; **blue: LDH-02;** and **red**: **Cu**_2_ **O@LDH-01**).

For the **Cu**_**2**_ **O@LDH-01** sample, an additional band around 1100 cm^–1^ is attributed to Cu–O stretching vibrations from Cu_2_O species dispersed on the LDH surface and to layered Cu–OH vibrations observed in **LDH-02**.^29^ Moreover, the absorption peaks near 550 and 400 cm^–1^ are assigned to δ_HO–M–OH_ and δ_O–M–O_ bending vibrations, respectively, where M represents Mg, Cu, or Al. These results collectively confirm the coexistence of hydrotalcite-like structural units and supported Cu_2_O nanoparticles within the composite system.

Nitrogen adsorption–desorption analyses provided valuable insights into how the distinct copper oxidation states in the catalysts influence their textural and catalytic properties. Both **Cu**_**2**_ **O@LDH-01** and **LDH-2** exhibit comparable surface areas of approximately 89 m^2^ g^–1^, characteristic of mesoporous materials suitable for catalytic applications; however, their adsorption behaviors reveal notable differences in the nature of their active sites.

For **Cu**_**2**_ **O@LDH-01**, a moderate *C* constant of 165.8, combined with excellent linearity (*R* ^2^ = 0.99994) over the measured pressure range (*P*/*P*_0_ = 0.054–0.299), indicates the presence of Cu­(I) sites that interact with nitrogen molecules in a manner favorable for catalytic processes involving π-backbonding interactions. This observation is consistent with the catalyst intended application in alkyne activation for click chemistry, where such electronic interactions are critical for the formation of the copper-acetylide intermediate.

In contrast, **LDH-2** exhibits significantly stronger adsorption characteristics, as evidenced by a higher *C* constant (196.5) and a substantial nitrogen uptake (15.95 cm^3^ g^–1^) at very low relative pressures (*P*/*P*_0_ = 0.013). The monolayer capacity (*Q*_m_) was determined using the molecular cross-sectional area (σ) of nitrogen, allowing for the calculation of the BET surface area, the results of which are summarized in Table 1.

**Table 1:** Surface Area (*S*_BET_), Monolayer Capacity (*Q*_m_), and Molecular Cross-Sectional Area (σ)

catalyst	*S*_BET_ (m^2^ g^–1^)	*Q*_m_ (m^3^ g^–1^)	σ (nm^2^)
Cu_2_O@LDH-01	89.50	20.56	0.162
LDH-2	89.85	20.64	0.162

The observed adsorption behavior indicates the presence of highly polar, potentially charged active sites, consistent with exposed Cu­(II) species, which could facilitate electrophilic processes. The pronounced differences in the low-pressure adsorption isotherms of the two materials, despite their comparable surface areas, highlight the critical influence of copper oxidation state on the surface adsorptive properties.^30^

The multilayer adsorption observed in the isotherms is consistent with the mesoporous nature of both materials. These distinct textural characteristics correlate with their divergent catalytic performances. The surface of **Cu**_**2**_ **O@LDH-01** appears particularly suited for CuAAC reactions, likely due to their enhanced π-backbonding capability, which is crucial for terminal alkyne activation. In contrast, the predominantly electrophilic character of **LDH-2**, while less favorable for cycloaddition, suggests potential utility in oxidation catalysis, representing an avenue for future investigation.^30^

Taken together, the XRD, FTIR, and BET analyses establish a clear structure–activity relationship, demonstrating that the copper oxidation state is a key determinant of surface reactivity and, consequently, catalytic application. These insights not only rationalize the observed catalytic performance but also provide a predictive framework for the rational design of copper-based catalysts tailored to specific transformations.^17^

Scanning transmission electron microscopy (STEM)-coupled EDX further reveals the morphology of the prepared catalysts (Figure 3). Both **LDH-2** (Figure 3A) and **Cu**_**2**_ **O@LDH-01** (Figure 3B) exhibit the characteristic plate-like morphology of LDHs. In addition, SEM images of **Cu**_**2**_ **O@LDH-01** confirm the presence of the Cu_2_O nanoparticulate phase, which is uniformly distributed across the surface of the **LDH-01** support, ensuring the accessibility of the catalytic sites.

**Figure 3:**
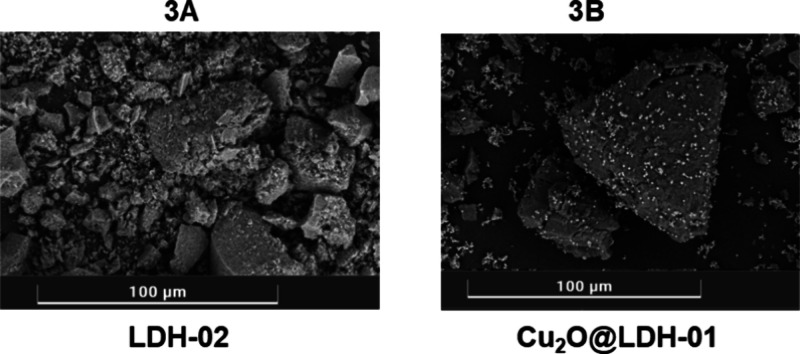
SEM images of the catalysts: (A) **LDH-02** and (B) **Cu**_2_ **O@LDH-01**.

EDX analysis (Figure 4) provides both qualitative and quantitative information about the elemental composition of the catalysts. As summarized in Table 2, the EDX results are consistent with the theoretically predicted composition, confirming the successful incorporation of the expected elements into the LDH-supported Cu_2_O nanocatalysts.

**Figure 4:**
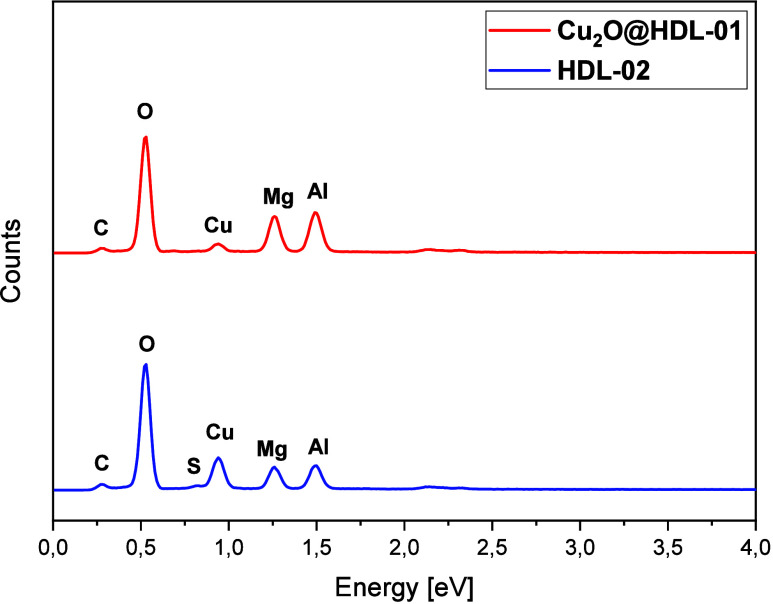
EDX spectra.

**Table 2:** Elemental Composition of Catalysts from EDX

	samples			
	LDH-02		Cu_2_O@LDH-01	
element	atomic %	weight %	atomic %	weight %
oxygen	54.41	46.29	50.27	48.97
carbon	28.27	18.05	29.68	20.75
aluminum	5.38	7.72	8.48	13.32
magnesium	5.70	7.37	7.93	11.22
copper	5.93	20.02	1.63	6.04
sulfur	0.32	0.54		

The presence of sulfur in the **LDH-02** catalyst originates from the synthesis procedure, which employs copper­(II) sulfate as the Cu^2+^ source, resulting in residual sulfate anions retained within the LDH interlayers.

The distinct surface properties revealed by BET analysis correlate directly with the elemental distribution observed in SEM images (Figure 3), where the uniform dispersion of copper species (Cu^+^ in **Cu**_**2**_ **O@LDH-01** versus Cu^2+^ in **LDH-2**) visually confirms the heterogeneous yet well-defined active sites responsible for the adsorption behavior of each material. As noted by Yu et al.,^23^ the interaction between Cu_2_O and the support is critical to prevent nanoparticle aggregation and oxidation. In our study, the **LDH-01** support exhibited a similar effect, ensuring a high Cu_2_O dispersion and sustained catalytic activity. These results reinforce that the observed homogeneous dispersion of Cu_2_O particles arises from strong interactions with the support.^14^

TGA of the nanocomposites was performed in air (50 mL min^–1^) over the temperature range of 30–600 °C with a heating rate of 10 °C min^–1^ (Figure 5). Both samples exhibited a typical lamellar decomposition behavior characteristic of hydrotalcites.^27^ An initial weight loss below 100 °C (∼16% by mass) is attributed to the removal of surface hydroxyl groups and physically adsorbed water. The second stage, occurring below 420 °C, involves a mass loss of approximately 17%, corresponding to the dehydroxylation of the layers and partial decomposition of the interlayer carbonate ions. At temperatures above 545 °C, a further mass loss of ∼4.9% is observed, attributed to complete decarboxylation and collapse of the lamellar structure, resulting in the formation of mixed oxides.

**Figure 5:**
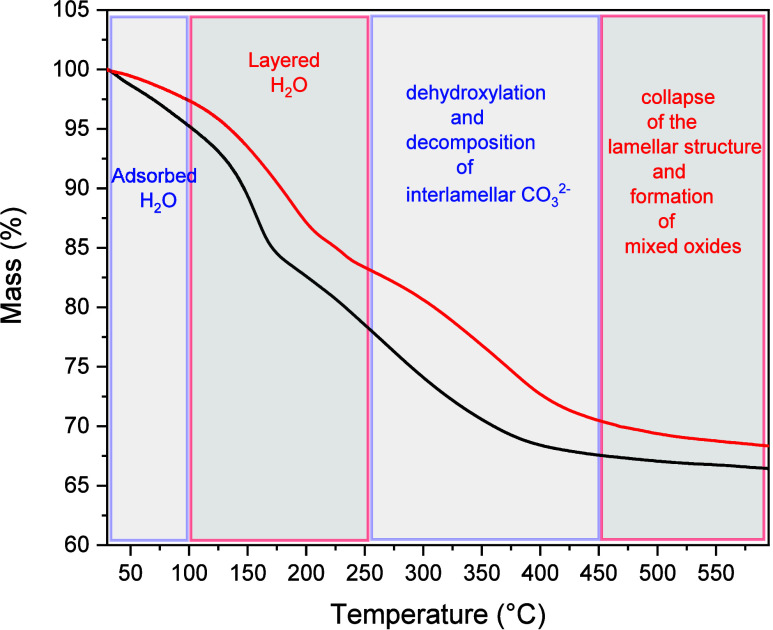
TGA graph of the nanocomposites (**black**: **LDH-02;** **red**: **Cu**_2_ **O@LDH-01**).

Following structural and textural characterization, the catalytic performance of the materials was evaluated in the model CuAAC reaction.

### Catalytic Effect

The catalytic parameters were optimized using the reaction between phenylacetylene (**1**) and benzylazide (**2**) as a model transformation (Table 3). Phenylacetylene was selected due to its relatively high terminal acidity, which facilitates the formation of the key copper-acetylide intermediate and its widespread use as a benchmark substrate in CuAAC studies.^1,8,21^ Benzylazide was chosen as a stable, commercially available partner with minimal steric and electronic effects, allowing the intrinsic activity of the catalyst toward alkyne activation to be the main factor governing reaction efficiency.^31,32^ The use of a preformed azide also simplifies the reaction setup and prevents side reactions commonly associated with in situ azide generation protocols.^2^

**Table 3:**
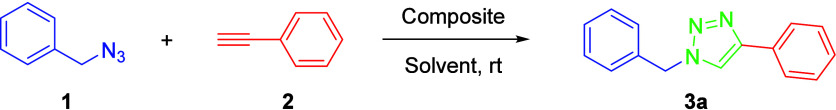
Reaction Optimization Study for CuAAC Coupling^a^,^b^

entry	composite	catalyst loading (wt %)	time (h)	solvent	conversion (%)^c^
1	Cu_2_O@LDH-01	15	24	EtOH	77
2	LDH-02	15	24	EtOH	17
3	LDH-01	15	24	EtOH	
4	Cu_2_O@LDH-01	15	24	CH_3_CN	33
5	LDH-02	15	24	CH_3_CN	51
6	Cu_2_O@LDH-01	15	24	CHCl_3_	16
7	LDH-02	15	24	CHCl_3_	40
8	Cu_2_O@LDH-01	15	24	*t*-BuOH	63
9	LDH-02	15	24	*t*-BuOH	45
10	Cu_2_O@LDH-01	15	24	DMSO	77
11	LDH-02	15	24	DMSO	88
12	Cu_2_O@LDH-01	15	24	H_2_O	100
13	LDH-02	15	24	H_2_O	100
14	Cu_2_O@LDH-01	15	24	DMF	98
15	LDH-02	15	24	DMF	95
16	Cu_2_O@LDH-01	15	24	MeOH	100
17	LDH-02	15	24	MeOH	100
18	Cu_2_O@LDH-01	15	24	THF	53
19	LDH-02	15	24	THF	30
20	Cu_2_O@LDH-01	6.5	1	H_2_O	100
21	LDH-02	6.5	1	H_2_O	99
22	Cu_2_O@LDH-01	6.5	12	MeOH	100
23	LDH-02	6.5	12	MeOH	95
24	Cu_2_O@LDH-01	6.5	5 min^d^	H_2_O	100
25	LDH-02	6.5	5 min^d^	H_2_O	90
26	Cu_2_O@LDH-01	6.5	5 min^d^	MeOH	100
27	LDH-02	6.5	5 min^d^	MeOH	95

a Reaction conditions: **1** (1.0 mmol), **2** (1.2 equiv), catalyst as indicated, rt, solvent volume = 5 mL.

b Note: In water, both substrates form a heterogeneous mixture; however, the reaction proceeds efficiently due to rapid interfacial activation at the**Cu**_**2**_ **O@LDH** surface.

c Conversion relative to azide determined by ^1^H NMR.

d Ultrasound irradiation.

Optimization of the reaction conditions was performed through 27 experimental entries, systematically varying the solvent, reaction time, catalyst loading, catalyst type, and physical conditions (ultrasound). Entry 3, employing copper-free **LDH-01**, served as a control and confirmed the essential role of copper species in activating the catalytic system.

The solvent effect was investigated using representative polar protic, polar aprotic, and nonpolar media, and the observed reactivity trends were correlated to solvent polarity using the dielectric constant (ε) as a quantitative descriptor. In polar protic solvents such as water (entries 12, 13, 16, 17; ε = 80.0) and methanol (entries 20, 21; ε = 33.0), both catalysts achieved quantitative conversion (100%) even at reduced catalyst loading (6.5 wt %).

Remarkably, entries 24 and 25, which employed ultrasonic irradiation, exhibited a significant enhancement in efficiency: acoustic cavitation promoted superior catalyst dispersion and intensified mass transfer, affording nearly 100% conversion within only 5 min in aqueous medium. The drastic rate acceleration observed under ultrasound is consistent with literature precedents,^19,20,22^ where ultrasonic energy was shown to improve dispersion and catalytic turnover in CuAAC reactions. This underscores the key role of physical activation methods in enhancing mass transfer and catalyst accessibility in heterogeneous systems.^11,18^

The excellent performance of heterogeneous copper catalysts in water was previously demonstrated by Prasad et al.,^18^ who reported quantitative conversions under mild conditions, emphasizing the stabilizing effect of the hydrotalcite matrix on Cu­(I) species. Our results with **Cu**_**2**_ **O@LDH-01** in aqueous medium (100% conversion) align with these findings, further suggesting that the synergy between solvent polarity and the porous LDH structure is crucial for catalytic efficiency.^8,12^ In addition, ultrasonic irradiation not only enhances mass transfer but also induces continuous mechanical depassivation of the catalyst surface, ensuring that active copper sites remain exposed and accessible to the reactants.^33^

In polar aprotic solvents such as DMSO (entries 10 and 11; ε = 46.7) as well as DMF (entries 14 and 15; ε = 38.0), high conversions were obtained. This efficiency can be attributed to the ability of these solvents to coordinate with metal centers and stabilize transition states without compromising the nucleophilicity of the azide. These observations are consistent with the findings of Alonso et al.^1^ and Amini et al.,^2^ who reported excellent catalytic performance of CuNPs and Cu­(I) species in polar aprotic media, particularly within heterogeneous systems.

In moderately polar protic solvents such as ethanol (ε = 24.6; entries 1, 2) and *tert*-butanol (ε = 12.5; entries 8, 9), conversions ranged from moderate to good (17–77% with Cu­(I)), reflecting a delicate balance between substrate solubility, catalyst–substrate interaction, and solvent polarity sufficient to stabilize the transition state. Notably, ethanol showed strong compatibility with **Cu**_**2**_ **O@LDH-01** (entry 1, 77%) but significantly lower activity with **LDH-02** (entry 2, 17%). This contrast likely arises from the requirement of Cu­(I) species for efficient copper-acetylide formation, as proposed by Namitharan et al.^3^

The moderate efficiency observed in solvents of intermediate polarity such as ethanol parallels the results reported by Chetia et al.,^9^ who employed hydrotalcite-supported CuNPs in ethylene glycol. Although ethylene glycol afforded high yields, the **Cu**_**2**_ **O@LDH-01** system demonstrated superior versatility under aqueous conditions, underscoring the stabilizing role of the LDH matrix toward the Cu­(I) oxidation state. Furthermore, the higher performance of Cu­(I) species (**Cu**_**2**_ **O@LDH-01**) in less polar solvents such as acetonitrile aligns with previous reports,^9,10,12^ which attribute this effect to the facilitated formation of copper-acetylide intermediates at less coordinated surface sites.

Nonpolar or low-polarity solvents, such as chloroform (ε = 4.81; entries 6, 7) and THF (ε = 7.6; entries 18, 19), exhibited limited catalytic performance, with conversions ranging from 16 to 53%. The low dielectric constants of these solvents hamper the solvation of polar intermediates and restrict reagent diffusion toward the active sites. A similar behavior has been reported by Dar et al.^21^ and Amini et al.^2^ for Cu­(II)-based heterogeneous systems supported on clays, emphasizing the crucial role of the reaction medium in determining the catalytic efficiency.

A comparative assessment between **Cu**_**2**_ **O@LDH-01** (Cu­(I)) and **LDH-02** (Cu­(II)) further demonstrates that, although both catalysts perform effectively in highly polar media, Cu­(I) species display superior activity in solvents of intermediate or low polarity, such as acetonitrile (ε = 37.5; entries 4, 5) and *tert*-butanol (ε = 12.5; entries 8, 9). This trend suggests reduced competition with coordinating solvents and enhanced accessibility of active sites, in agreement with the observations of Yagmurlu et al.^34^ in homogeneous Cu­(I) systems.

The BET isotherm C constant of **Cu**_**2**_ **O@LDH-01** (165.8) supports this interpretation, indicating a moderate adsorption energy sufficient to anchor reactants without hindering catalytic turnover. The superior performance of **Cu**_**2**_ **O@LDH-01** (Cu­(I)) in solvents of intermediate polarity, such as acetonitrile and ethanol, also aligns with recent findings that highlight the stabilization of Cu­(I) sites through electronic interactions with layered or carbonaceous supports.^23^ These interactions not only preserve the active oxidation state but also modulate the electron density at the copper centers, thereby facilitating the formation of the key copper-acetylide intermediate essential to the click reaction mechanism.

Interestingly, **Cu**_**2**_ **O@LDH-01**, featuring copper in the +1 oxidation state on the surface, traditionally regarded as the most active form for copper–acetylide formation, did not consistently outperform **LDH-02**, which contains Cu^2+^ species within a layered framework. This behavior, observed across multiple solvent systems, underscores that catalytic performance arises from a complex interplay among the oxidation state, copper site localization, in situ redox processes, and the structural stability of the matrix.

The synergy between active-site accessibility and catalyst stability, evidenced by the BET analysis, aligns with the findings of Hu et al.,^10^ who reported a Cu­(I)-containing MOF capable of promoting one-pot aqueous reactions. Their study emphasized the critical role of controlled porosity (ca. 12 Å) in sustaining catalytic efficiency, an insight directly translatable to our LDH-based system.

Therefore, the efficiency of heterogeneous CuAAC in liquid media depends not only on the solvent dielectric constant, which governs the solubility and stabilization of reactive intermediates, but also on key physical properties of the system such as dispersion, mass transfer, and accessibility of active sites. These parameters can be further optimized through physical activation techniques such as ultrasonic irradiation, which enhance interfacial contact and sustain the exposure of catalytically active copper centers.^14,17,30,33^

The results demonstrate that the catalytic performance of **Cu2O@LDH-01** is governed by a delicate interplay among solvent polarity, copper oxidation state, and physicochemical dynamics of the system, with BET data offering crucial insights into active-site accessibility. The measured surface area of 89.5 m^2^ g^–1^ and a C constant of 165.8 reveal a moderately high specific surface populated by Cu­(I) sites capable of π-backbonding interactions, an essential feature for efficient alkyne activation in CuAAC.

In highly polar solvents (water, methanol; ε > 30), quantitative conversions (∼100%) reflect not only the stabilization of polar intermediates such as copper-acetylide but also the excellent accessibility of catalytic sites, as corroborated by the BET isotherm exhibiting multilayer adsorption at intermediate pressures (*P*/*P*_0_ = 0.054–0.299). The high activity observed in DMSO and DMF (95–98%), despite their known ability to coordinate Cu­(I), is attributed to the mesoporous nature of the catalyst, which maintains azide nucleophilicity while balancing the solvation and diffusion effects. The moderate surface area of **Cu**_**2**_ **O@LDH-01** (89.5 m^2^ g^–1^) lies between those reported by Anvari et al. (133 m^2^ g^–1^)^8^ and Hu et al. (45.1 m^2^ g^–1^),^10^ suggesting an optimal balance between active-site density and substrate diffusion.

In contrast, low-polarity solvents such as chloroform and THF (ε < 10) yielded significantly reduced conversions (30–40%), underscoring the dependence of catalytic activity on efficient solid–liquid interfacial interactions. The low dielectric constant hinders penetration of reactants into the porous network, even in the presence of energetically favorable Cu­(I) sites (*C* = 165.8).^30^ Remarkably, ultrasonic irradiation overcame this limitation, achieving complete conversion within 5 min by inducing acoustic cavitation that disrupts aggregates, exposes the internal porosity, and enhances mass transfer throughout the catalytic matrix.^33^

The comparative analysis between **Cu**_**2**_ **O@LDH-01** (Cu­(I)) and **LDH-02** (Cu­(II)), the latter characterized by a higher BET C constant (196.5), indicative of more electrophilic Cu­(II) sites, reveals distinct catalytic behaviors governed by the solvent polarity. While both catalysts exhibit high activity in polar media, Cu­(I)-containing **Cu**_**2**_ **O@LDH-01** consistently outperforms **LDH-02** in less polar solvents, such as acetonitrile. This trend aligns with the BET results: the lower *C* constant of **Cu**_**2**_ **O@LDH-01** (165.8) reflects a weaker adsorption energy, minimizing competition for coordination with aprotic solvents and thereby facilitating rapid formation of the copper-acetylide intermediate. In contrast, **LDH-02** displays strong nitrogen uptake at very low relative pressures (15.95 cm^3^ g^–1^ at *P*/*P*_0_ = 0.013), suggesting highly polar, strongly adsorbing Cu­(II) sites that are more prone to solvent coordination, ultimately restricting their catalytic versatility. These findings are consistent with previous studies demonstrating that Cu­(I)-containing hydrotalcites achieve superior performance in azide–alkyne cycloaddition due to enhanced site accessibility and balanced interactions with polar media.^15,18^

Water proved to be the optimal solvent, not only maximizing the interaction with the heterogeneous catalyst but also simplifying product isolation, thereby reinforcing the sustainability of the process.^6,7^ The use of water and benign substrates thus supports the development of a robust, versatile, and industrially relevant catalytic protocol, ideally suited for benchmarking the activity of composites such as **Cu**_**2**_ **O@LDH-01** and **LDH-02** under diverse operational conditions.^16^

This optimized catalyst architecture combines electronically accessible Cu­(I) sites (BET: *C* = 165.8), structural stability confirmed by XRD and TGA, and full compatibility with aqueous media, establishing **Cu**_**2**_ **O@LDH-01** as a superior platform for regioselective triazole synthesis. Moreover, the system provides a solid foundation for expanding its application scope to a wide range of alkynes and azides under the identified green and efficient reaction conditions.

### Substrate Structure–Reactivity Relationships

The results obtained for the substrate scope reactions carried out in an aqueous medium using the heterogeneous catalyst **Cu**_**2**_ **O@LDH-01** reveal a clear correlation between the physicochemical properties within the series of compounds studied and the corresponding reaction yields (Scheme 1).

**Scheme 1:**
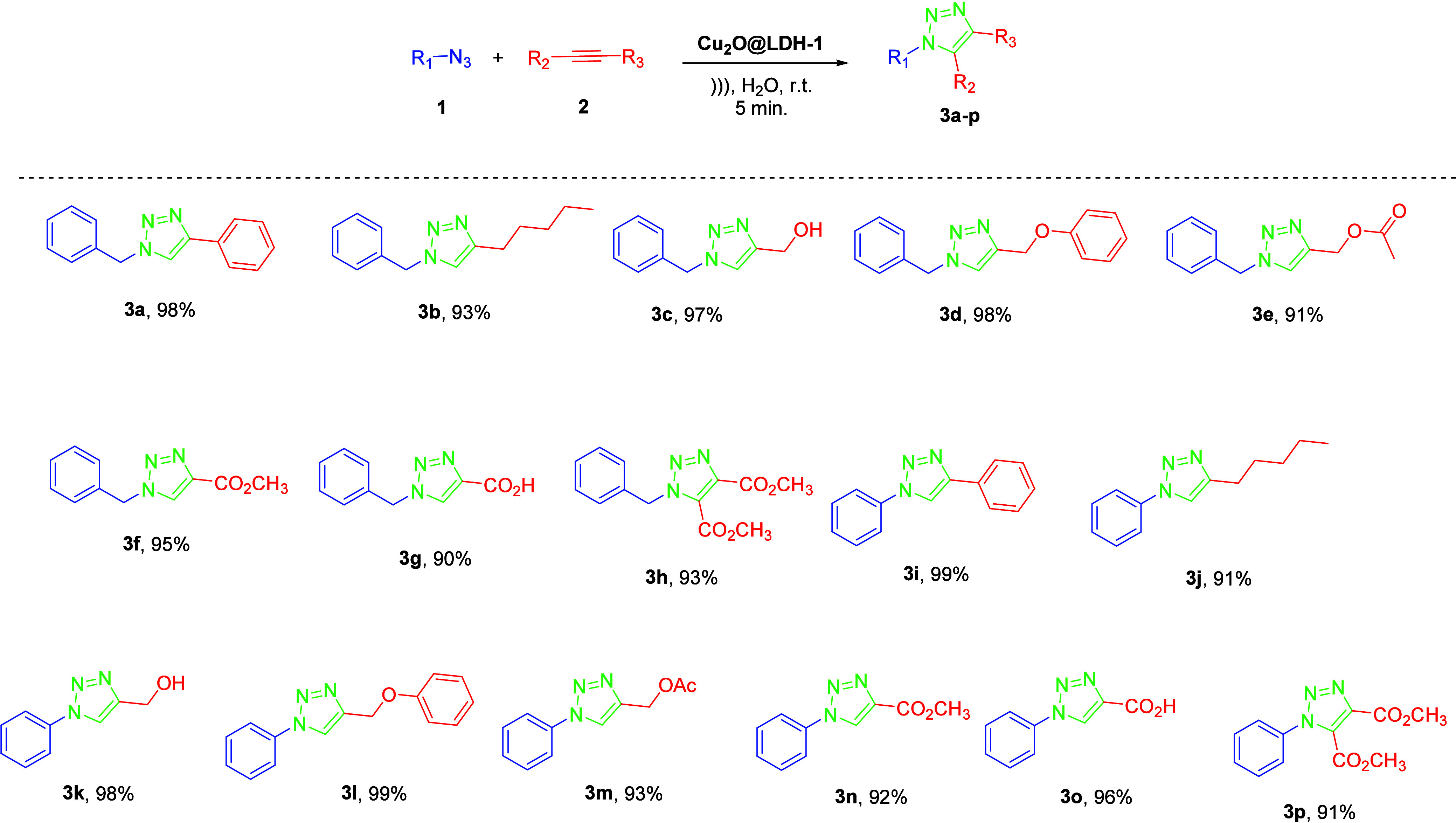
Scope of 1,2,3-Triazoles **3a**–**p** ^a^

a Reaction conditions: azide (1.0 mmol), alkyne (1.0 mmol), and catalyst (6.5 mol wt%) at 25 °C. Isolated yield after simple filtration. In some cases, the reaction time was 1 h for **3c**, **3d**, and **3e** or 3 h for **3b** and **3g.**

Analysis of the azide–alkyne cycloaddition (CuAAC) catalyzed by the **Cu**_**2**_ **O@LDH-01** system reveals a pronounced dependence of the substrate reactivity on the electronic, steric, and interfacial factors that govern their interaction with the catalytic centers. With the confirmed robustness of the catalyst and the stability of Cu­(I) as the active species, it becomes evident that the reaction efficiency is intrinsically linked to the nature of both the alkyne and azide substrates.

Electron-deficient alkynes, such as methyl propiolate, propiolic acid, and dimethyl but-2-ynedioate, displayed quantitative conversions when reacted with benzylazide, highlighting the catalyst’s remarkable ability to activate the C≡C bond. The presence of electron-withdrawing groups in these substrates likely facilitates the formation of the Cu-acetylide intermediate, a key step in the CuAAC mechanism as described by Himo et al.^35^ This trend, previously observed by Amini et al.^2^ and Yu et al.,^23^ appears to be further enhanced in the present system by the electronic modulation exerted by the hydrotalcite support, which tunes the electron density around Cu­(I) and promotes an optimal catalytic environment.

The choice of azide proved to be decisive in several cases. Benzylazide and phenylazide, although structurally analogous, yielded markedly different results when reacted with less reactive alkynes. In the case of benzylazide, hept-1-yne afforded low conversion (20% after 5 min), whereas with phenylazide, the yield increased dramatically to quantitative levels (100%). This contrast can be attributed to the planar geometry and electronically deficient nature of phenylazide, which promote tighter molecular packing at the catalytic interface and more favorable initial coordination with Cu­(I). The aromatic conjugation and reduced conformational flexibility of phenylazide likely facilitate π–π and Cu–N interactions, compensating for solubility limitations or steric hindrance of the alkyne.^36^ These observations, unreported in systems such as those described by Chetia et al.,^9^ underscore the specific and often underestimated role of the azide in modulating CuAAC reactivity.

Conversely, alkynes containing potentially coordinating functional groups, such as hydroxyl or ether moieties, exhibited reduced yields when reacted with benzylazide. Prop-2-yn-1-ol and (prop-2-yn-1-yloxy)­benzene displayed significantly higher conversions in the presence of phenylazide, suggesting that benzylazide may engage in competitive coordination with Cu­(I) centers or with the basic hydrotalcite surface, partially inhibiting the process.^37^ These results indicate that substrate functional compatibility with the catalyst surface, particularly in aqueous media, must be carefully considered when designing future CuAAC transformations involving multifunctional alkynes.

A notable turning point emerged in the study of dimethyl but-2-ynedioate, an activated internal alkyne, which afforded quantitative conversion with benzylazide but only 34% with phenylazide. This inversion underscores fundamental mechanistic differences between terminal and internal alkynes in CuAAC. The absence of a terminal hydrogen precludes conventional activation via deprotonation, rendering the reaction more sensitive to the steric orientation and spatial compatibility between the reacting partners. The rigid, planar geometry of phenylazide can impose steric hindrance when approaching the internal alkyne, whereas the greater conformational flexibility of benzylazide facilitates effective docking and transition-state formation. This subtle mechanistic feature has been scarcely explored in the literature and rarely addressed in heterogeneous CuAAC studies,^1,2^ highlighting the novelty and significance of the present observations.

The high performance of the **Cu**_**2**_ **O@LDH-01** catalyst can also be attributed to its mesoporous architecture (89.5 m^2^ g^–1^) and the high accessibility of Cu­(I) active sites (BET *C* constant = 165.8), which ensure efficient catalysis even with bulky or less polar substrates. The hydrophilic–hydrophobic interface of the catalyst further promotes the selective diffusion of reactants, optimizing their interaction with active sites. Compared to the system reported by Anvari et al.,^8^ which operates in aqueous medium at elevated temperatures (50 °C) with reaction times up to 20 min, **Cu**_**2**_ **O@LDH-01** demonstrates superior versatility, selectivity, and reactivity under milder, environmentally friendly conditions.

The operational stability and reusability of **Cu**_**2**_ **O@LDH-01** were evaluated by using the model reaction between benzylazide and phenylacetylene. The catalyst was successfully recycled and reused for five consecutive cycles without any significant loss of activity (Figure 6). After each run, the catalyst was recovered by simple filtration, washed with dichloromethane, and directly reused under the standard reaction conditions (water, room temperature, 5 min, ultrasound irradiation, 6.5 wt % catalyst). These results highlight the robustness of **Cu**_**2**_ **O@LDH-01** and confirm its effectiveness as a truly heterogeneous and recyclable system for the CuAAC reaction.^38,39^

**Figure 6:**
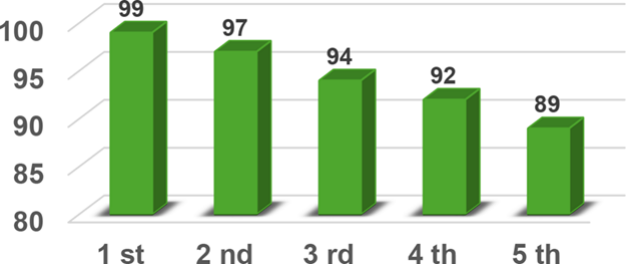
Recyclability of the catalyst.

In contrast to carbon- or silica-supported catalysts, as well as homogeneous systems that often require more stringent conditions,^12^ **Cu**_**2**_ **O@LDH-01** efficiently promotes the CuAAC reaction in aqueous media at room temperature, highlighting its alignment with the principles of green chemistry. The exceptional performance observed even with challenging substrates indicates that the hydrotalcite-supported solid–liquid interface plays a critical role in both solubilizing the reactants and orienting them effectively at the active sites.

Overall, these findings demonstrate that successful heterogeneous CuAAC in water arises from a synergistic combination of electronic activation, steric effects, functional group compatibility, and interactions at the catalyst–substrate interface. **Cu**_**2**_ **O@LDH-01** emerges not only as a sustainable and highly efficient catalytic platform but also as a model system for guiding the rational design of substrates and reaction conditions in modern organic synthesis. As emphasized by Gawande et al.,^12^ the future development of heterogeneous Cu­(I) catalysis hinges on supports that simultaneously offer structural stability, active-site accessibility, and compatibility with green solvents.

## Conclusions

In conclusion, we have developed a robust heterogeneous catalyst, **Cu**_**2**_ **O@LDH-01**, which significantly enhances the practicality and sustainability of the CuAAC reaction. The strategic immobilization of cuprous oxide nanoparticles within the mesoporous hydrotalcite framework was crucial in creating a truly heterogeneous system, effectively preventing copper leaching and ensuring exceptional operational stability. The synergy between this advanced material design and ultrasound irradiation is particularly notable, enabling quantitative conversions in aqueous media within an unprecedented 5 min time frame, thus establishing a new benchmark for efficiency in this class of transformations.

Beyond its outstanding catalytic performance, the system maintains consistent activity over five consecutive recycling cycles, highlighting its industrial viability. The structure–activity relationships elucidated herein provide valuable molecular-level insights into the interplay of electronic and steric factors at the active sites, offering a predictive framework for future reaction optimization. This work transcends the development of a single catalyst; it presents a blueprint for designing next-generation earth-abundant metal catalysts on layered supports. Accordingly, **Cu**_**2**_ **O@LDH-01** represents a versatile and sustainable platform for the rapid and green synthesis of 1,2,3-triazoles, with broad implications for drug discovery, materials science, and process chemistry.

The demonstrated efficiency, stability, and operational simplicity of **Cu**_**2**_ **O@LDH-01** provide a compelling foundation for future scale-up studies and potential applications in continuous-flow processes.

Future studies will focus on expanding the substrate scope to include alkyl azides, unsymmetrical internal alkynes, and substrates bearing more sensitive functional groups to further delineate the limits of this catalytic system.

## Experimental Section

### Materials and Methods

#### Chemistry

The reagents were purchased from Sigma-Aldrich Brazil and were used without further purification. Powder XRD data were collected using a Bruker D8 Advance DaVinci diffractometer with CuKα radiation, LynxEye XE-T Linear Position Sensitive Detector, Ni filter, and Bragg–Brentano geometry. Data were collected between 8° and 80° in 2θ with a step size of 0.02° and a count time of 0.5 s per step. A Soller slit with 272.5° divergence and a 0.2 mm divergent slit were used. TEM and EDX images were obtained using a JEOL, JSM 5610LV transmission electron microscope operated at 10 keV. The indicated yields refer to homogeneous materials purified by filtration and confirmed by spectroscopic techniques. Melting points were obtained on a Thermo Scientific 9100 apparatus and were uncorrected. The specific surface areas (BET) of the catalysts were determined by using a Micromeritics ASAP 2420 instrument. The samples were pretreated at 100 °C for 24 h, followed by an in situ treatment under reduced pressure at 250 °C, with a heating rate of 5 °C min^–1^, for 24 h. After treatment, N_2_ physisorption was performed at −196 °C. Infrared spectra were collected by using KBr pellets on a PerkinElmer model 1420 FT-IR spectrophotometer, and the spectra were calibrated relative to the 1601.8 cm^–1^ absorbance of polystyrene. ^1^H nuclear magnetic resonance (NMR) spectra were recorded at room temperature (rt) using a Bruker AVANCE NEO 500 MHz, in CDCl_3_ or DMSO-*d*_6_. The chemical shift data were reported in units of δ (ppm) downfield from solvent, and the solvent was used as an internal standard; coupling constants (*J*) are reported in hertz and refer to apparent peak multiplicities.

#### Catalyst Preparation

##### Synthesis of LDHs

The preparation of the three LDHs was performed by the coprecipitation method with the use of a ratio of divalent metallic cations.^25^ 0.5 L of an aqueous solution of NaOH (10.16 g, 254 mmol, 6.35 equiv) and Na_2_CO_3_ (2.69 g, 25.4 mmol, 0.65 equiv) was slowly added to 0.5 L of an aqueous solution containing aluminum nitrate salt (15.00 g, 40 mmol of Al­(NO_3_)_3_·9H_2_O, 1 equiv) and the other salts (80 mmol (20.50 g, 2 equiv) of Mg­(NO_3_)_2_·6H_2_O for **LDH-01**; 40 mmol of Mg­(NO_3_)_2_·6H_2_O (10.25 g, 1 equiv) and 40 mmol (9,98 g, 1 equiv) of CuSO_4_·5H_2_O for **LDH-02**) and stirred at room temperature for 24 h. The precipitate was then filtered and washed with distilled water until a neutral pH was obtained.

##### Synthesis of Supported Nano-Cu_2_O in MgAl-LDH

The preparation of the Cu_2_O nano-supported catalyst (**Cu**_**2**_ **O@LDH-01**) was performed by a method adapted from the literature.^9,26^ First, hydrotalcite (5.0 g) was dispersed with CuSO_4_·5H_2_O (0.5 g) in water (500 mL) at room temperature for 30 min. Hydrazine hydrate (50%, 10 mL) was added dropwise for 30 min, and the reaction mixture was stirred at room temperature for 3 h. The obtained solid was centrifuged and washed with distilled water and acetone to remove unreacted reagents. The product was dried at 100 °C for 8 h and stored in desiccators.

##### Synthesis of Benzylazide

To a stirred solution of sodium azide (3.40 g, 52.1 mmol, 2.0 equiv) in 80 mL of acetone–water (3:1 v/v) mixture at room temperature was added benzyl chloride (3.3 g (3.0 mL), 26.1 mmol, 1.0 equiv) and stirred further until completion (thin-layer chromatography, TLC) of reaction in 12 h.^40^ The reaction mixture was diluted with 30 mL of water and extracted with 150 mL of dichloromethane. The aqueous layer was extracted with (2 × 25 mL) of dichloromethane. The combined organic layers were dried over anhydrous Na_2_SO_4_ and then concentrated under reduced pressure to yield pure benzylazide **1** as a pale-yellow oil in 95% yield (3.31 g). Characterization by NMR and IR spectroscopy confirmed the purity, and the compound was used in subsequent reactions without further purification.

##### Synthesis of Phenylazide

To a stirred solution of aniline (1.86 g, 20 mmol, 1.0 equiv) in aqueous HCl (4 mL of conc. HCl in 22 mL of H_2_O) at 0 °C was added a solution of sodium nitrite (1.52 g, 22 mmol, 1.1 equiv) in H_2_O (6 mL) dropwise, maintaining the internal temperature below 5 °C. The mixture was stirred for 20 min at 0 °C before the dropwise addition of a solution of sodium azide (1.43 g, 22 mmol, 1.1 equiv) in H_2_O (8 mL). The reaction mixture was stirred for 1 h, at which point TLC analysis (30% EtOAc/hexane) indicated complete consumption of the starting material. The mixture was extracted with EtOAc (3 × 50 mL). The combined organic layers were washed with sat. NaHCO_3_ (3 × 25 mL) and brine (1 × 25 mL), dried over anhydrous Na_2_SO_4_, filtered, and concentrated under reduced pressure to afford the title compound.

##### Protocol for Reaction Optimization

The reactions were conducted using phenylacetylene (0.5 mmol) as the model alkyne and benzylazide (**1**, 0.5 mmol), with catalyst loadings varying between 15 mg (entries 1–19) and 6.5 mg (entries 20–25), both corresponding to approximately 6.5 and 15% by mass relative to the azide, respectively, in 5 mL of appropriate solvent at room temperature. Reaction progress was monitored by TLC, and conversions were quantified by ^1^H NMR after catalyst removal and solvent evaporation.

##### General Procedure for the Synthesis of 1,4-Disubstituted-1*H*-1,2,3-triazole (**3a–p**)

To a mixture of azide **1** (1 mmol, 1 equiv) and alkyne **2** (1.1 mmol, 1.1 equiv) in water (3 mL), the catalyst (6.5 mg, 6.5 wt %) was added. The mixture was stirred at room temperature until the completion of the reaction, which was confirmed by TLC. After completion of the reaction, it was filtered, and the products were characterized by ^1^H spectroscopic data without further purification.

##### 1-Benzyl-4-phenyl-1*H*-1,2,3-triazole (**3a**)

White crystal solid, mp 129–132 °C (lit.^9^ 128–131 °C), 98% yield. ^1^H NMR (500 MHz, CDCl_3_) δ 7.69 (d, *J* = 7.3 Hz, 1H), 7.57 (s, 1H), 7.32–7.24 (m, 2H), 7.18 (ddd, *J* = 13.2, 7.7, and 5.8 Hz, 2H), 5.44 (s, 1H).

##### 1-Benzyl-4-butyl-1*H*-1,2,3-triazole (**3b**)

Pale-yellow solid, mp 43–45 °C (lit.^41^ 42 °C), 93% yield. ^1^H NMR (500 MHz, CDCl_3_) δ: 7.43–7.30 (m, 3H), 7.28–7.16 (m, 3H), 5.50 (s, 2H), 2.68 (s, 2H), 1.66 (s, 2H), 1.29 (m, 4H), 0.98 (s, 3H).

##### (1-Benzyl-1*H*-1,2,3-triazol-4-yl) Methanol (**3c**)

White solid, mp 76.77 °C (lit.^42^ 76–78 °C), 97% yield. ^1^H NMR (500 MHz, CDCl_3_) δ 7.73 (s, 1H), 7.44–7.35 (m, 5H), 7.28 (d, *J* = 4.4 Hz, 3H), 5.53 (s, 2H), 4.85 (s, 2H).

##### 1-Benzyl-4-(phenoxymethyl)-1*H*-1,2,3-triazole (**3d**)

White solid, mp 120–123 °C (lit.^42^ 119–121 °C), 98% yield. ^1^H NMR (500 MHz, CDCl_3_) δ: 7.56 (s, 1H), 7.47–7.37 (m, 3H), 7.35–7.26 (m, 4H), 7.03–6.94 (m, 3H), 5.56 (s, 2H), 5.22 (s, 2H).

##### (1-Benzyl-1*H*-1,2,3-triazol-4-yl)­methyl Acetate (**3e**)

White crystal solid, mp 56–57 °C (lit.^43^ 58–59 °C), 91% yield. ^1^H NMR (500 MHz, CDCl_3_) δ 7.58 (s, 1H), 7.41–7.34 (m, 3H), 7.28 (dd, *J* = 8.4 and 6.7 Hz, 2H), 5.51 (s, 2H), 5.17 (s, 2H), 2.04 (s, 3H).

##### Methyl 1-Benzyl-1*H*-1,2,3-triazole-4-carboxylate (**3f**)

Off-white solid, mp 115–117 °C (lit.^42^ 116–118 °C), 95% yield. ^1^H NMR (500 MHz, CDCl_3_) δ 7.93 (s, 1H), 7.29 (dd, *J* = 5.2 and 1.9 Hz, 2H), 7.20 (dd, *J* = 7.1 and 2.3 Hz, 3H), 5.48 (s, 2H), 3.82 (s, 3H).

##### 1-Benzyl-1*H*-1,2,3-triazole-4-carboxylic Acid (**3g**)

Brown solid, mp 178–179 °C (lit.^44^ 177–179 °C), 90% yield. ^1^H NMR (500 MHz, DMSO-*d*_6_) δ: 7.33 (s, 1H), 6.52–6.43 (m, 6H), 5.05 (s, 2H).

##### Dimethyl 1-Benzyl-1*H*-1,2,3-triazole-4,5-dicarboxylate (**3h**)

Pale-brown crystals, mp 47–48 °C (lit.^45^ 45–46 °C), 90% yield. ^1^H NMR (500 MHz, CDCl_3_) δ 7.29–7.23 (m, 1H), 7.19 (dd, *J* = 7.5 and 4.9 Hz, 1H), 5.73 (s, 1H), 3.88 (s, 1H), 3.80 (d, *J* = 8.4 Hz, 1H).

##### 1-(4-Diphenyl)-1*H*-1,2,3-triazole (**3i**)

Pale-yellow solid, mp 183–184 °C (lit.^46^ 185–185.5 °C), 98% yield. ^1^H NMR (500 MHz, CDCl_3_) δ 8.24 (s, 1H), 7.95 (d, *J* = 7.3 Hz, 2H), 7.82 (d, *J* = 7.7 Hz, 2H), 7.57 (t, *J* = 7.3 Hz, 2H), 7.48 (m, 3H), 7.40 (t, *J* = 7.1 Hz, 1H).

##### 4-Butyl-1-phenyl-1*H*-1,2,3-triazole (**3j**)

Yellow oil (lit.^47^), 95% yield. ^1^H NMR (500 MHz, CDCl_3_) δ 7.76 (s, 1H), 7.73 (d, *J* = 7.8 Hz, 1H), 7.51 (t, *J* = 7.7 Hz, 1H), 7.42 (t, *J* = 7.4 Hz, 1H), 2.80 (t, *J* = 7.7 Hz, 1H), 1.80–1.70 (m, 1H), 1.38 (d, *J* = 3.4 Hz, 2H), 0.91 (t, *J* = 6.8 Hz, 2H).

##### (1-Phenyl-1*H*-[1,2,3]­triazol-4-yl)-methanol (**3k**)

White solid, mp 115–118 °C (lit.^48^ 110–111 °C), 98% yield. ^1^H NMR (500 MHz, CDCl_3_) δ 8.08 (s, 1H), 7.73 (d, *J* = 7.9 Hz, 2H), 7.54 (t, *J* = 7.7 Hz, 2H), 7.46 (t, *J* = 7.4 Hz, 1H), 4.93 (s, 2H), 2.86 (s, 2H).

##### 4-(Phenoxymethyl)-1-phenyl-1*H*-1,2,3-triazole (**3l**)

White solid, mp 81–83 °C (lit.^49^ 80–81 °C), 98% yield. ^1^H NMR (500 MHz, CDCl_3_) δ 8.08 (s, 1H), 7.77 (d, *J* = 7.7 Hz, 2H), 7.56 (t, *J* = 7.9 Hz, 2H), 7.48 (t, *J* = 7.4 Hz, 1H), 7.34 (ddd, *J* = 7.0, 6.1, and 3.4 Hz, 3H), 7.09–6.98 (m, 3H), 5.34 (s, 2H).

##### (1-Phenyl-1*H*-1,2,3-triazol-4-yl)­methyl Acetate (**3m**)

White solid, mp 53–55 °C (lit.^48^ 54–55 °C), 95% yield. ^1^H NMR (500 MHz, CDCl_3_) δ 8.10 (s, 1H), 7.76 (d, *J* = 7.9 Hz, 2H), 7.56 (t, *J* = 7.7 Hz, 3H), 7.48 (t, *J* = 7.4 Hz, 1H), 5.32 (s, 2H), 2.12 (d, *J* = 15.4 Hz, 4H).

##### Methyl 1-Phenyl-1*H*-1,2,3-triazole-4-carboxylate (**3n**)

Yellow oil (lit.^50^), 97% yield. ^1^H NMR (500 MHz, CDCl_3_) δ 8.57 (s, 1H), 7.78 (d, *J* = 7.9 Hz, 2H), 7.59 (t, *J* = 7.7 Hz, 3H), 7.55–7.50 (m, 1H), 4.03 (s, 3H).

##### 1-Phenyl-1*H*-1,2,3-triazole-4-carboxylic Acid (**3o**)

White solid, mp 90–92 °C (lit.^51^ 89–91 °C), 96% yield. ^1^H NMR (500 MHz, DMSO-*d*_6_) δ 9.31 (s, 1H), 7.93 (d, *J* = 7.9 Hz, 2H), 7.60 (t, *J* = 7.8 Hz, 2H), 7.55–7.49 (m, 2H).

##### 1-Phenyl-1*H*-[1,2,3]­triazole-4,5-dicarboxylic Acid Dimethyl Ester (**3p**)

White solid, mp 105–109 °C (lit.^51^ 106–108 °C), 92% yield. ^1^H NMR (500 MHz, CDCl_3_) δ: 7.61–7.54 (m, 5H), 4.03 (s, 3H), 3.94 (s, 3H).

## Supplementary Material



## Notes

Details of the complete experimental procedures, characterization data, and NMR spectra (PDF)
