# Supplementary material for: Recyclable Hydrotalcite-Supported Copper Catalysts for Green and Regioselective Click Synthesis of 1,2,3-Triazoles
Source: ACS Omega. 2026 Jan 27;11(5):8249–59. doi: 10.1021/acsomega.5c10849 (PMC12903169; doi:10.1021/acsomega.5c10849)
Supplement: Supplementary file 1 [file ao5c10849_si_001.pdf]

Recyclable Hydrotalcite-Supported Copper Catalysts for Green and Regioselective Click Synthesis of 1,2,3-Triazoles

Gustavo S. G. de Carvalho,* Douglas C. A. Pinto, Fernando de C. da Silva**

Universidade Federal Fluminense, Instituto de Química, Departamento de Química Orgânica, Campus do Valonguinho, Niterói-RJ, Brazil.

E-mail: senradcarvalho@gmail.com*; fcsilva@id.uff.br**

Keywords: Click chemistry, CuACC reaction, heterogenous catalysis, layered double hydroxide.

Contents

I. EXPERIMENTAL
II. ANALYTICAL DATA
III. REFERENCES

I. EXPERIMENTAL

Catalysts preparation

Synthesis of Layered Double Hydroxides

The preparation of the three LDHs was performed by the co-precipitation method with the use of a ratio of divalent metallic cations.[1] 0.5 L of an aqueous solution of NaOH (10.16 g, 254 mmol, 6.35 equiv.) and Na_2CO_3 (2.69 g, 25.4 mmol, 0.65 equiv.) was slowly added to 0.5 L of an aqueous solution containing aluminum nitrate salt (15.00 g, 40 mmol of $Al(NO_3)_3$•$9H_2O$, 1 equiv.) and the other salts (80 mmol (20.50 g, 2 equiv.) of $Mg(NO_3)_2$•$6H_2O$ for **LDH-01**; 40 mmol of $Mg(NO_3)_2$•$6H_2O$ (10.25 g, 1 equiv.) and 40 mmol (9,98 g, 1 equiv.) of $CuSO_4$•$5H_2O$ for **LDH-02**) and stirred at room temperature for 24 h. The precipitate was then filtrated and washed with distilled water until a neutral pH was obtained.

Synthesis of supported nano Cu_2O in MgAl-LDH

The preparation of Cu_2O nano supported catalyst (**Cu_2O@LDH-01**) was performed by a method adapted from literature.[2,3] First, hydrotalcite (5.0 g) was dispersed with $CuSO_4 \cdot 5H_2O$ (0.5 g) in water (500 mL) at room temperature for 30 min. Hydrazine hydrate (50%, 10 mL) was added drop wise for 30 min and the reaction mixture was stirred at room temperature for 3 h. The obtained solid was filtered and washed with distilled water and acetone, to remove un-reacted reagents. The product was dried at 100 °C for 8 h and stored in desiccators.

Synthesis of Benzylazide

To a stirring solution of sodium azide (3.40 g, 52.1 mmol, 2.0 equiv.) in 80 mL of acetone-water (3:1 v/v) mixture at r.t. was added benzyl chloride (3.3 g (3.0 mL), 26.1 mmol, 1.0 equiv.) and was stirred further until completion (TLC) of reaction in 12 h.[4] The reaction mixture was diluted with 30 mL of water and extracted with 150 mL of dichloromethane. The aqueous layer was extracted with (2 x 25 mL) of dichloromethane. The combined organic layers were dried over anhydrous Na_2SO_4 and then concentrated under reduced pressure to yield pure benzylazide **1** as a pale-yellow oil in 95.5% yield (3.31 g). ^1H NMR (500 MHz in $CDCl_3$): δ 7.36 (m, 5H), 4.33 (s, 2H, CH_2).

Synthesis of Phenylazide

To a stirred solution of aniline (1.86 g, 20 mmol, 1.0 equiv.) in aqueous HCl (4 mL of conc. HCl in 22 mL of H_2O) at 0 °C was added a solution of sodium nitrite (1.52 g, 22 mmol, 1.1 equiv.) in H_2O (6 mL) dropwise, maintaining the internal temperature below 5 °C. The mixture was stirred for 20 min at 0 °C before the dropwise addition of a solution of sodium azide (1.43 g, 22 mmol, 1.1 equiv.) in H_2O (8 mL). The reaction was stirred for 1 h, at which point TLC analysis (30% EtOAc/hexanes) indicated complete consumption of the starting material. The mixture was extracted with EtOAc (3 x 50 mL). The combined organic layers were washed with sat. aq. $NaHCO_3$ (3 x 25 mL) and brine (1 x 25 mL), dried over

anhydrous Na_2SO_4, filtered, and concentrated under reduced pressure to afford the title compound.

Protocol to reactions optimization

The reactions were conducted using phenylacetylene (0.5 mmol) as model alkyne and benzylazide (0.5 mmol), with catalyst loadings varying between 15 mg (entries 1 to 19) and 6.5 mg (entries 20 to 25), both corresponding to approximately 6.5 and 15% by mass relative to the azide, respectively, in 5 mL of appropriate solvent at room temperature. Reaction progress was monitored by thin layer chromatography (TLC), and conversions were quantified by ^1H NMR after catalyst removal and solvent evaporation.

II. ANALYTICAL DATA

General procedure for the synthesis of 1,4-disubstituted-1H-1,2,3-triazole

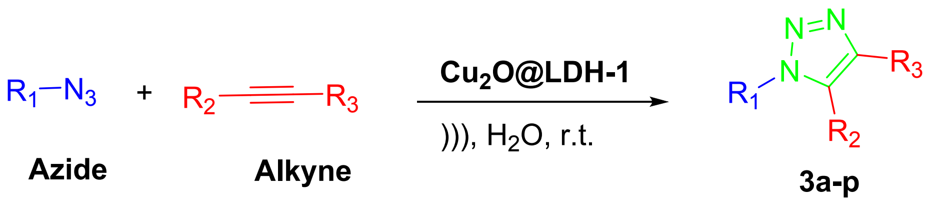

To a mixture of azide **1** (1 mmol, 1 equiv.) and acetylene **2** (1.1 mmol, 1.1 equiv.) in water (3 mL), the catalyst (6.5 mg, 6.5 wt%) was added. The mixture was stirred at room temperature till the completion of the reaction which was confirmed by TLC. After completion of the reaction, it was filtered, and the products were characterized by ^1H spectroscopic data without further purification.

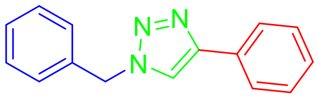

1-Benzyl-4-phenyl-1H-1,2,3-triazole (3a). White crystal solid, mp 129-132 °C (lit.[2] 128-131 °C), 98% yield. ^1H NMR (500 MHz, $CDCl_3$) δ 7.69 (d, J = 7.3 Hz,

1H), 7.57 (s, 1H), 7.32 – 7.24 (m, 2H), 7.18 (ddd, J = 13.2, 7.7, 5.8 Hz, 2H), 5.44 (s, 1H).

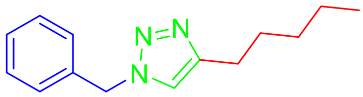

1-Benzyl-4-butyl-1H-1,2,3-triazole (3b). Yellow pale solid, mp 43-45 °C (lit.[5] 42 °C), 93% yield. ^{1}H NMR (500 MHz, $CDCl_3$) δ 7.43 – 7.30 (m, 3H), 7.28 – 7.16 (m, 3H), 5.50 (s, 2H), 2.68 (s, 2H), 1.66 (s, 2H), 1.29 (m, 4H), 0.98 (s, 3H).

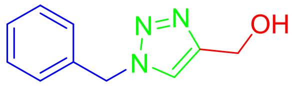

(1-Benzyl-1H-1,2,3-triazol-4-yl) methanol (3c). White solid, mp 76.77 °C (lit.[6] 76-78 °C), 97% yield. ^{1}H NMR (500 MHz, $CDCl_3$) δ 7.73 (s, 1H), 7.44 – 7.35 (m, 5H), 7.28 (d, J = 4.4 Hz, 3H), 5.53 (s, 2H), 4.85 (s, 2H).

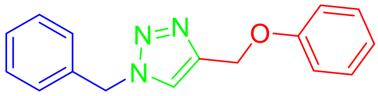

1-benzyl-4-(phenoxymethyl)-1H-1,2,3-triazole (3d). White solid, mp 120-123 °C (lit.[6] 119-121 °C), 98% yield. ^{1}H NMR (500 MHz, $CDCl_3$) δ 7.56 (s, 1H), 7.47 – 7.37 (m, 3H), 7.35 – 7.26 (m, 4H), 7.03 – 6.94 (m, 3H), 5.56 (s, 2H), 5.22 (s, 2H).

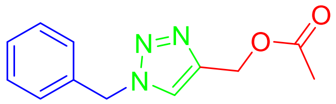

(1-benzyl-1H-1,2,3-triazol-4-yl)methyl acetate (3e). White crystal solid, mp 56-57 °C (lit.[7] 58-59 °C), 91% yield. ^{1}H NMR (500 MHz, $CDCl_3$) δ 7.58 (s, 1H), 7.41 – 7.34 (m, 3H), 7.28 (dd, J = 8.4, 6.7 Hz, 2H), 5.51 (s, 2H), 5.17 (s, 2H), 2.04 (s, 3H).

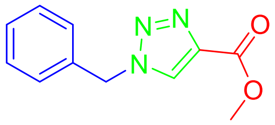

methyl 1-benzyl-1H-1,2,3-triazole-4-carboxylate (3f). White-off solid, mp 115-117 °C (lit.[6] 116-118 °C), 95% yield. ^{1}H NMR (500 MHz, $CDCl_3$) δ 7.93 (s, 1H), 7.29 (dd, *J* = 5.2, 1.9 Hz, 2H), 7.20 (dd, *J* = 7.1, 2.3 Hz, 3H), 5.48 (s, 2H), 3.82 (s, 3H).

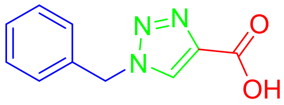

1-benzyl-1H-1,2,3-triazole-4-carboxylic acid (3g). Brown solid, mp 178-179 °C (lit.[8] 177-179 °C), 90% yield. ^{1}H NMR (500 MHz, DMSO-d_6) δ 7.33 (s, 1H), 6.52 – 6.43 (m, 6H), 5.05 (s, 2H).

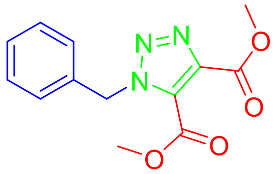

dimethyl 1-benzyl-1H-1,2,3-triazole-4,5-dicarboxylate (3h). Brown pale crystals, mp 47-48 °C (lit.[9] 45-46 °C), 90% yield. ^{1}H NMR (500 MHz, $CDCl_3$) δ 7.29 – 7.23 (m, 1H), 7.19 (dd, *J* = 7.5, 4.9 Hz, 1H), 5.73 (s, 1H), 3.88 (s, 1H), 3.80 (d, *J* = 8.4 Hz, 1H).

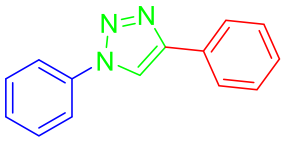

1-(4-Diphenyl)-1H-1,2,3-triazole (3i). Pale yellow solid, mp 183-184 °C (lit.[10] 185-185.5 °C), 98% yield. ^{1}H NMR (500 MHz, $CDCl_3$) δ 8.24 (s, 1H), 7.95 (d, *J* = 7.3 Hz, 2H), 7.82 (d, *J* = 7.7 Hz, 2H), 7.57 (t, *J* = 7.3 Hz, 2H), 7.48 (m, 3H), 7.40 (t, *J* = 7.1 Hz, 1H).

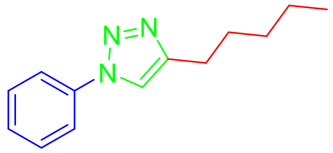

4-Butyl-1-phenyl-1H-1,2,3-triazole (3j). Yellow oil (lit.[11]), 95% yield. ^{1}H NMR (500 MHz, $CDCl_3$) δ 7.76 (s, 1H), 7.73 (d, *J* = 7.8 Hz, 1H), 7.51 (t, *J* = 7.7 Hz, 1H), 7.42 (t, *J* = 7.4 Hz, 1H), 2.80 (t, *J* = 7.7 Hz, 1H), 1.80 – 1.70 (m, 1H), 1.38 (d, *J* = 3.4 Hz, 2H), 0.91 (t, *J* = 6.8 Hz, 2H).

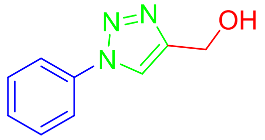

(1-Phenyl-1H-[1,2,3]triazol-4-yl)-methanol (3k). White solid, mp 115-118 °C (lit.[12] 110-111 °C), 98% yield. ^{1}H NMR (500 MHz, $CDCl_3$) δ 8.08 (s, 1H), 7.73 (d, *J* = 7.9 Hz, 2H), 7.54 (t, *J* = 7.7 Hz, 2H), 7.46 (t, *J* = 7.4 Hz, 1H), 4.93 (s, 2H), 2.86 (s, 2H).

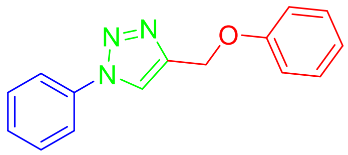

4-(phenoxymethyl)-1-phenyl-1H-1,2,3-triazole (3l). White solid, mp 81-83 °C (lit.[13] 80-81 °C), 98% yield. ^{1}H NMR (500 MHz, $CDCl_3$) δ 8.08 (s, 1H), 7.77 (d, *J* = 7.7 Hz, 2H), 7.56 (t, *J* = 7.9 Hz, 2H), 7.48 (t, *J* = 7.4 Hz, 1H), 7.34 (ddd, *J* = 7.0, 6.1, 3.4 Hz, 3H), 7.09 – 6.98 (m, 3H), 5.34 (s, 2H).

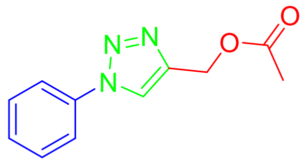

(1-phenyl-1H-1,2,3-triazol-4-yl)methyl acetate (3m). White solid, mp 53-55 °C (lit.[12] 54-55 °C), 95% yield. ^{1}H NMR (500 MHz, $CDCl_3$) δ 8.10 (s, 1H), 7.76 (d, *J* = 7.9 Hz, 2H), 7.56 (t, *J* = 7.7 Hz, 3H), 7.48 (t, *J* = 7.4 Hz, 1H), 5.32 (s, 2H), 2.12 (d, *J* = 15.4 Hz, 4H).

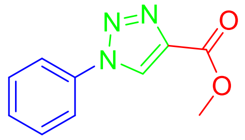

methyl 1-phenyl-1H-1,2,3-triazole-4-carboxylate (3n). Yellow oil (lit.[14]), 97% yield. ^{1}H NMR (500 MHz, $CDCl_3$) δ 8.57 (s, 1H), 7.78 (d, *J* = 7.9 Hz, 2H), 7.59 (t, *J* = 7.7 Hz, 3H), 7.55 – 7.50 (m, 1H), 4.03 (s, 3H).

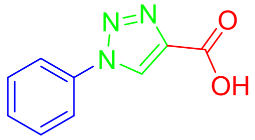

1-phenyl-1H-1,2,3-triazole-4-carboxylic acid (3o). White solid, mp 90-92 °C (lit.[15] 89-91 °C), 96% yield. ^{1}H NMR (500 MHz, DMSO-d6) δ 9.31 (s, 1H), 7.93 (d, *J* = 7.9 Hz, 2H), 7.60 (t, *J* = 7.8 Hz, 2H), 7.55 – 7.49 (m, 2H).

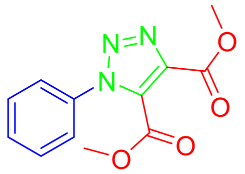

1-Phenyl-1H-[1,2,3]triazole-4,5-dicarboxylic acid dimethyl ester (3p). White solid, mp 105-109 °C (lit.[15] 106-108 °C), 92% yield. ^{1}H NMR (500 MHz, $CDCl_3$) δ 7.61 – 7.54 (m, 5H), 4.03 (s, 3H), 3.94 (s, 3H).

III. REFERENCES

(1) De Carvalho, G. S. G.; Chagas, L. H.; Fonseca, C. G.; De Castro, P. P.; Sant'Ana, A. C.; Leitão, A. A.; Amarante, G. W. Nb_2O_5 Supported on Mixed Oxides Catalyzed Oxidative and Photochemical Conversion of Anilines to Azoxybenzenes. *New J. Chem.* **2019**, *43* (15), 5863–5871. https://doi.org/10.1039/C9NJ00625G.

(2) Chetia, M.; Singh Gehlot, P.; Kumar, A.; Sarma, D. A Recyclable/Reusable Hydrotalcite Supported Copper Nano Catalyst for 1,4-Disubstituted-1,2,3-Triazole Synthesis via Click Chemistry Approach. *Tetrahedron Lett.* **2018**, *59* (4), 397–401. https://doi.org/10.1016/j.tetlet.2017.12.051.

(3) Song, X.; Xu, W.; Su, D.; Tang, J.; Liu, X. The Synthesis of Hollow/Porous Cu_2O Nanoparticles by Ion-Pairing Behavior Control. *ACS Omega* **2020**, *5* (4), 1879–1886. https://doi.org/10.1021/acsomega.9b03380.

(4) Rono, C. K.; Darkwa, J.; Meyer, D.; Makhubela, B. C. E. A Novel Series of N-Aryltriazole and N-Acridinyltriazole Hybrids as Potential Anticancer Agents. *Curr. Org. Synth.* **2019**, *16* (6), 900–912. https://doi.org/10.2174/1570179416666190704112904.
(5) Shin, J.-A.; Lim, Y.-G.; Lee, K.-H. Copper-Catalyzed Azide–Alkyne Cycloaddition Reaction in Water Using Cyclodextrin as a Phase Transfer Catalyst. *J. Org. Chem.* **2012**, *77* (8), 4117–4122. https://doi.org/10.1021/jo3000095.
(6) Girard, C.; Önen, E.; Aufort, M.; Beauvière, S.; Samson, E.; Herscovici, J. Reusable Polymer-Supported Catalyst for the [3+2] Huisgen Cycloaddition in Automation Protocols. *Org. Lett.* **2006**, *8* (8), 1689–1692. https://doi.org/10.1021/ol060283l.
(7) Sun, S.; Wu, P. Mechanistic Insights into Cu(I)-Catalyzed Azide−Alkyne “Click” Cycloaddition Monitored by Real Time Infrared Spectroscopy. *J. Phys. Chem. A* **2010**, *114* (32), 8331–8336. https://doi.org/10.1021/jp105034m.
(8) Maisonial, A.; Serafin, P.; Traïkia, M.; Debiton, E.; Théry, V.; Aitken, D. J.; Lemoine, P.; Viossat, B.; Gautier, A. Click Chelators for Platinum-Based Anticancer Drugs. *Eur. J. Inorg. Chem.* **2008**, *2008* (2), 298–305. https://doi.org/10.1002/ejic.200700849.
(9) El Hajjaji, S.; Lgamri, A.; Aziane, D.; Guenbour, A.; Essassi, E. M.; Akssira, M.; Ben Bachir, A. Synthesis and Evaluation of the Inhibitor Effect of a New Class of Triazole Compounds. *Prog. Org. Coat.* **2000**, *38* (3–4), 207–212. https://doi.org/10.1016/S0300-9440(00)00102-8.
(10) Revathi, S.; Ghatak, T. N-Heterocyclic Imine-Supported Bimetallic Cu(II) Catalyst for Azide-Alkyne Cycloaddition: Solvent-free, Reductant-free, Ppm-level Catalysis to Access 1,4-Disubstituted Triazoles. *Chem. – Asian J.* **2023**, *18* (10), e202300156. https://doi.org/10.1002/asia.202300156.
(11) Kolarovič, A.; Schnürch, M.; Mihovilovic, M. D. Tandem Catalysis: From Alkynoic Acids and Aryl Iodides to 1,2,3-Triazoles in One Pot. *J. Org. Chem.* **2011**, *76* (8), 2613–2618. https://doi.org/10.1021/jo1024927.
(12) Gonzaga, D.; Senger, M. R.; Da Silva, F. D. C.; Ferreira, V. F.; Silva, F. P. 1-Phenyl-1H- and 2-Phenyl-2H-1,2,3-Triazol Derivatives: Design, Synthesis and Inhibitory Effect on Alpha-Glycosidases. *Eur. J. Med. Chem.* **2014**, *74*, 461–476. https://doi.org/10.1016/j.ejmech.2013.12.039.
(13) Jiang, Y.; Kong, D.; Zhao, J.; Zhang, W.; Xu, W.; Li, W.; Xu, G. A Simple, Efficient Thermally Promoted Protocol for Huisgen-Click Reaction Catalyzed by CuSO4·5H2O in Water. *Tetrahedron Lett.* **2014**, *55* (15), 2410–2414. https://doi.org/10.1016/j.tetlet.2014.02.108.
(14) Li, W.; Zhou, X.; Luan, Y.; Wang, J. Direct Access to 1,4-Disubstituted 1,2,3-Triazoles through Organocatalytic 1,3-Dipolar Cycloaddition Reaction of α,β-Unsaturated Esters with Azides. *RSC Adv.* **2015**, *5* (108), 88816–88820. https://doi.org/10.1039/C5RA19038J.
(15) Garg, A.; Ali, A. A.; Damarla, K.; Kumar, A.; Sarma, D. Aqueous Bile Salt Accelerated Cascade Synthesis of 1,2,3-Triazoles from Arylboronic Acids. *Tetrahedron Lett.* **2018**, *59* (45), 4031–4035. https://doi.org/10.1016/j.tetlet.2018.09.064.
